# Determination of Viscoelastic Property in Polyethylene Crystallization Using a Quartz Crystal Resonator

**DOI:** 10.3390/s91209544

**Published:** 2009-11-30

**Authors:** Yong Kweon Suh, Byoung Chul Kim, Young Han Kim

**Affiliations:** 1 Department of Mechanical Engineering, Dong-A University, 840 Hadan-dong, Pusan, 604-714 Korea; E-Mail: yksuh@dau.ac.kr; 2 Department of Chemical Engineering, Dong-A University, 840 Hadan-dong, Pusan, 604-714 Korea; E-Mail: kbc1010@hanmail.net

**Keywords:** quartz crystal resonator, viscoelasticity, quartz mechanics, polymer crystallization

## Abstract

A new generalized relationship between the viscoelastic properties of an overlayer placed on the electrode interface of a quartz crystal resonator and its resonant characteristic is developed from the mechanics of the quartz movement. The relationship is used to estimate the viscoelastic properties from the experimentally measured resonant characteristic. It is utilized in the estimation of viscosity and elastic shear modulus of a polyethylene overlayer during its crystallization. The measurements are compared with the viscosity and elastic shear modulus of a polyethylene melt measured using a rheometer. It is found that the development of this study is useful in the determination of viscoelastic property of polymer materials by measuring the resonant frequency and conductance of the polymer overlayer placed on the resonator electrode.

## 1. Introduction

The variation of viscoelastic properties of a polymer material during its crystallization is important information in the design of a variety of polymer processing equipments. For instance, the property change has a key role in the design of a mold for an injection molding machine, but the property measurement is difficult while the polymer crystallizes.

A quartz crystal resonator is composed of a thin quartz crystal sandwiched between two metal electrodes that establish an alternating electric field across the crystal, causing vibrational motion of the crystal. The motion is characterized with the resonant frequency and admittance of the resonator, and the characteristic is sensitive to the changes of mass and physical property of an overlayer on its electrode. Because the resonator is so sensitive, it can sense a variety of changes in micro-scale at the electrode interface. In polymerization, the rheological properties of a reactant and product mixture vary continuously as the polymerization proceeds. The resonator has been implemented in the monitoring of a UV photopolymerization by measuring its resonant resistance [1]. The nucleation and crystal formation in a cooling crystallization have also been investigated with the quartz crystal resonator [2,3].

The resonant frequency and admittance of the resonator can be interpreted to the changes of mass and viscoelasticity of an overlayer at its electrode interface. Reed *et al.* [4] presented a physical description of a viscoelastically loaded resonator for either very thick viscous fluids or very thin elastic overlayers. Buttry and Ward [5] gave an extensive review of the quart crystal resonator including basic principles of an AT-cut resonator and relation of the mechanical system composed of a spring, mass and a damper to the electrical circuit of resistance, inductance and capacitance. Nwankwo and Durning [6] provided an impedance analysis of the resonator for very thick viscoelastic fluids. Kim *et al.* [7] demonstrated the applicability of a specially designed resonator system to monitor the change of viscoelastic property of thin polymer films subjected to temperature variation. When a PMMA/PVAc blend was coated on the one side of the resonator, the resonant frequency decreased accompanying hysteresis with the increased temperature. Based on the resonant frequency variation and the resonant resistance change, they demonstrated that there are two transient phase transition temperatures between the temperatures of 20 °C and 80 °C. Kunze *et al.* [8] developed an extended sheet-contact model to describe the change of resonant frequency and the dissipation of very thin viscoelastic solids coated on the electrode. Efimov *et al.* [9] studied the sensitivity variation of the resonator due to the energy trapping. It was found that energy trapping was insignificant for the small amount of mass loading, but the energy trapping became dominant and an oscillation occurred only in the region of the loading with a large loading. Thermoresponsive viscoelastic property of hydrogel was monitored with the impedance variation of a quartz crystal resonator [10]. A continuum mechanics model was utilized in the analysis of continuous viscoelastic profiles of a liquid film [11]. The frequency shift of viscoelastic overlayer has been interpreted with the small-load approximation [12]. A new set of equations was derived from the complex frequency shift of polymer brushed, and was applied to analyze the dissipation data [13].

In this study a generalized relation between the resonant characteristics of a quartz crystal resonator and the rheological properties of an overlayer applied on the electrode surface are developed from the mechanics of the quartz movement. The elastic shear modulus and viscosity of a polyethylene overlayer are estimated from the relation and the experimentally obtained resonant frequency and conductance of the resonator. The results are compared with the bulk property of polyethylene melt measured with a rheometer.

## 2. Theoretical Analysis

**Figure 1.**
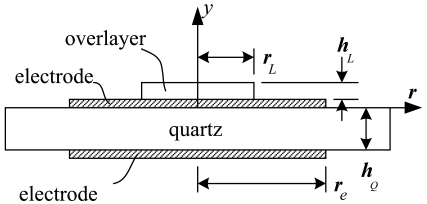
Sketch of a quartz crystal resonator with electrodes on both sides and a viscoelastic overlayer attached on the external surface of an electrode.

Consider the thickness-shear motion of a thin circular-disk-shape quartz crystal with thickness *h_Q_* having a pair of concentric electrodes with radius *r_e_* on both sides as shown in Figure 1.

The viscoelastic overlayer attached on the top electrode is assumed to be of axisymmetric shape with radius *r_L_* and thickness *h_L_*. Then the equation of motion for the quartz can be written as:

(1) $$c_{66}\frac{\partial^{2}u}{\partial y^{2}}+\eta_{Q}\frac{\partial^{3}u}{\partial t\partial y^{2}}+e_{26}\frac{\partial^{2}\phi }{\partial y^{2}}=\rho_{Q}\frac{\partial^{2}u}{\partial t^{2}}$$

(2) $$e_{26}\frac{\partial^{2}u}{\partial y^{2}}-{ɛ}_{22}\frac{\partial^{2}\phi }{\partial y^{2}}=0$$

where *t* is time and (*r, y*) denotes the radial and axial coordinates of the cylindrical coordinate system. Further, *u*(*r, y, t*) is the mechanical displacement of the quartz along the *x*-direction, ø(*r, y, t*) the electric potential, *c_66_* the elastic shear modulus of the quartz, *e*_26_ the piezoelectric constant of the quartz, ε_22_ the dielectric constant of the quartz, *η_Q_* the viscosity of the quartz, and *ρ_Q_* the volume density of the quartz material.

The equation for the viscoelastic overlayer is similar to Equation (1) but purely mechanical:

(3) $$\mu_{L}\frac{\partial^{2}\nu }{\partial y^{2}}+\eta_{L}\frac{\partial^{3}\nu }{\partial t\partial y^{2}}=\rho_{L}\frac{\partial^{2}\nu }{\partial t^{2}}$$

where *v*(*r, y, t*) is the mechanical displacement of the overlayer along the *x*-direction, *μ_L_* the elastic shear modulus, *η_L_* the viscosity, and *ρ_L_* the volume density.

We have six boundary conditions for the above set of equations. Firstly, no-slip condition must be satisfied at the interface between the quartz and the overlayer (through the intermediate thin electrode of course). Secondly, the shear stress should vanish on the top surface of the overlayer. Next, the potentials on the top and bottom electrode surfaces are specified from a specified AC field. The fifth and sixth boundary conditions can be given by applying the Newton's second law to the mass of the top and bottom electrodes, respectively. Here, the forces acting on the electrodes may include the shear forces from the quartz and/or the overlayer (only for the top electrode).

Solutions to Equations (1), (2) and (3) can be written as:

(4) $$u(r,y,t)=p(r)\overset{⌢}{u}(y)e^{i\omega t}$$

(5) $$\phi (r,y,t)=\left\{\left(e_{26}/{ɛ}_{22})p(r)\hat{u}(y)+\left[p(r)E-(2{\overset{⌢}{\phi }}_{0}/h_{Q})\right]y+F(r\right)\right\}e^{i\omega t}$$

(6) $$v(r,y,t)=p(r)\overset{⌢}{v}(y)e^{i\omega t}$$

where *ω* and *φ̂*_0_ the angular frequency and the amplitude of the external AC electric potential, respectively. The radial dependence of the displacements is represented by *p*(*r*) = exp(-*r^2^*/r_e_^2^). Further, we have:

(7) $$\overset{⌢}{u}=A\exp(ik_{Q}y)+B\exp(-ik_{Q}y)$$

(8) $$\overset{⌢}{v}=C\exp(ik_{L}y)+D\exp(-ik_{L}y)$$

where *k_Q_*=*ω*√(*ρ_Q_ /c̃*_66_) denotes the complex wave number and:

(9a) $${\tilde{c}}_{66}={\bar{c}}_{66}+i\omega \eta_{Q}$$

(9b) $${\bar{c}}_{66}=c_{66}+e_{26}^{2}/{ɛ}_{22}$$

The five unknown constants *A, B, C, D*, and *E* and one unknown function *F*(*r*) can be determined from the boundary conditions. After some algebra we arrive at the following formula for *E*:

(10) $$E=\frac{2{\overset{⌢}{\phi }}_{0}}{h_{Q}P_{e}}\frac{-K^{2}\left[2(1-\cos\psi_{Q})+2q_{e}\psi_{Q}+q_{L}\sin\psi_{Q}\right]}{\left(1-\cos\psi_{Q}+q_{e}\psi_{Q}\sin\psi_{Q}\right)\left(\psi_{Q}\cot(\psi_{Q}/2)-q_{e}\psi_{Q}^{2}-2K^{2}\right)+Q_{L}}$$

where:

$$Q_{L}=q_{L}\left[\psi_{Q}\cos\psi_{Q}-(q_{e}\psi_{Q}^{2}+K^{2})\sin\psi_{Q}\right]$$

$$K^{2}=\frac{e_{26}^{2}}{{ɛ}_{22}{\bar{c}}_{66}}$$

$$q_{e}=\frac{\rho_{e}}{\rho_{Q}h_{Q}}$$

$$q_{L}=\frac{\gamma k_{L}{\tilde{\mu }}_{L}\tan\psi_{L}}{k_{Q}{\bar{c}}_{66}}$$

$$\psi_{Q}=k_{Q}h_{Q}$$

$$\psi_{L}=k_{L}h_{L}$$

$${\tilde{\mu }}_{L}=\mu_{L}+i\omega \eta_{L}$$

$$\gamma =\frac{r_{L}^{2}P(r_{L})}{r_{e}^{2}P(r_{e})}$$

$$P(r)=\frac{1}{\pi r^{2}}\int_{0}^{r}p(r)2\pi \text{rdr}$$

and *ρ_e_* is the areal density of the electrode.

It can be shown that the admittance *Y*, defined as the ratio of amplitude of the current to that of the voltage applied across the electrodes, is given as:

(11) $$Y=i\omega C_{0}\left(1-\frac{h_{Q}P(r_{e})}{2{\overset{⌢}{\phi }}_{0}}E\right)$$

where *C_0_*=*ε_22_A_e_/h_Q_* is the static capacitance of quartz and *A_e_* is the area of the electrode. The admittance *Y* is composed of real part *G* and imaginary part *B* called conductance and susceptance, respectively.

The resonant frequency ƒ_0_ here is defined as the frequency at which *G* becomes the maximum. For the case of no overlayer (*q_L_* = *0*) and under the assumption of *K^2^* ≪ 1 and *q_e_* ≪ 1, the resonance frequency becomes:

(12) $$f_{0}=f_{00}\left[1-\left(4K^{2}/\pi^{2}+2q_{e}\right)\left(1-4q_{e}+2K^{2}q_{e}\right)\right]$$

where:

(13) $$f_{00}=1/\left(2h_{Q}\sqrt{\rho_{Q}/{\bar{c}}_{66}}\right)$$

It was shown by many investigators that the quartz crystal resonator can be understood in terms of an equivalent electrical circuit. Now, Equation (11) can be written as *Y* = *Y_0_*+*Y_m_*, where *Y_0_* = *iωC_0_* represents the admittance of the static capacitance *C_0_*, and *Y_m_* = –*iωC_0_h_0_P_e_*/(2*φ̂_0_*) denotes the admittance of motional branch. The impedance of the motional branch *Z_m_* is the inverse of *Y_m_*. Under the assumption that *ψ_Q_* = *k_Q_h_Q_* = *ψ(*1*-iξ)*, where *ψ* = *ωh_Q_*√(*ρ_Q_*/ c̄_66_), is very close to *π* for ƒ ≅ ƒ_0_ ≅ ƒ_00_, it can be shown that the impedance becomes:

$$Z_{m}=\frac{1}{8i\omega c_{0}K^{2}}\left\{\left(\pi^{2}-8K^{2})-[(1+4q_{e})\psi^{2}+2q_{LR}\psi ]+2i(\xi \psi^{2}+q_{LI}\psi \right)\right\}$$

Note that *ψ* represents the magnitude of *ψ_Q_*, and the ratio *ξ* is the imaginary part versus the real part of *ψ_Q_; ξ* = *ωη_Q_ /(2*c̄_66_). In this derivation, *ξ* is also assumed to be very small (*ξ* ≪ 1). Notice also that *q_LR_* and –*q_LI_* are the real and imaginary parts of *q_L_*, respectively: *q_L_* = *q_LR_* – *iq_LI_*. On the other hand the impedance *Z_m_* is assumed to be composed of resistance *R_m_*, capacitance *C_m_* and inductance *L_m_*:

$$Z_{m}=R_{m}+i\omega L_{m}+1/(i\omega C_{m})$$

Comparing these two, we get:

(14a) $$R_{m}=\frac{\pi \xi +q_{LI}}{8C_{0}K^{2}f_{0}}$$

(14b) $$L_{m}=\frac{1}{32C_{0}K^{2}f_{0}^{2}}\left[(1-8K^{2}/\pi^{2})+2q_{LR}/\pi \right]$$

(14c) $$C_{m}=\frac{8C_{0}K^{2}}{\pi^{2}-8K^{2}}$$

where ƒ_0_ is given as Equation (12). All the three formulas shown above are the same as those derived by Martin *et al.* [14].

The resonant frequency with the overlayer can be obtained from the three components derived above; ƒ_0L_=1/[2π√(*L_m_C_m_*)]. Therefore, the shift of resonant frequency Δ *ƒ_0L_* = *ƒ_0L_* – *ƒ_0_* becomes:

(15) $$\Delta f_{0L}=-\frac{q_{LR}}{\pi }f_{0}$$

On the other hand, following the R-L-C circuit theory, *G_max_* is given by:

(16) $$G_{\max}=\frac{1}{R_{m}}=\frac{8C_{0}K^{2}f_{0}}{\pi \xi +q_{LI}}$$

In addition, *G_max_*Δƒ_12_ is given as *G_max_*Δƒ_12_ =ƒ_0L_/(*QR_m_*), where Q = 2π*L_m_/R_m_* is the Q-factor, Δƒ_12_=ƒ_2_ – ƒ_1_ the band width, and ƒ_1_ and ƒ_2_ the values of the frequency at which the susceptance becomes the maximum and minimum, respectively. Then, we derive:

(17) $$G_{\max}\Delta f_{12}=\frac{1}{2\pi L_{m}}=\frac{16K^{2}{ɛ}_{22}A_{e}f_{0}^{2}}{\pi h_{Q}}$$

This indicates that *G_max_*Δƒ_12_ which is a measure of the static capacitance of the quartz is independent of the properties of the overlayer.

We can see from Equations (15) and (16) that two important parameters that reflect the properties of the material of the viscoelastic overlayer are Δƒ*_0L_* and *G_max_*; the magnitude of the former increases proportionally with the raised *q_LR_* and the latter decreases with the raised *q_LI_*. On the other hand, we have:

(18) $$q_{L}=M\exp(i\theta /2)\tan\left[N\exp(-i\theta /2)\right]$$

where:

(19a) $$M=\frac{\gamma \sqrt{\rho_{L}\mu }}{\sqrt{\rho_{Q}{\bar{c}}_{66}}},N=\frac{\pi h_{L}\sqrt{\rho_{L}/\mu }}{h_{Q}\sqrt{\rho_{Q}/{\bar{c}}_{66}}}$$

and:

(19b) $$\mu =\sqrt{\mu_{L}^{2}+{\left(\omega_{0}\eta_{L}\right)}^{2}},\theta =\tan^{-1}(\omega_{0}\eta_{L}/\mu_{L})$$

in the range of ***θ*** being 0 ≤ ***θ*** ≤ π/2.

For an effective analysis, we need to introduce two group variables, ∏_1_=*h_L_*√(c̄_66_ /(*h_Q_*√*μ)*) and ∏_2_ =ω_0_η*_L_/*μ*_L_*. We note that the asymptotically thin film of the material provides ∏_1_ → 0 and the asymptotically thick limit gives ∏_1_ → ∞. Similarly, a very hard material means ∏_2_ → 0 and the Newtonian liquid ∏_2_ → ∞. Further, in the following analysis we assume that *γ* = *O*(1) and *ρ_L_* ∼ *ρ_Q_*.

First, for the case of thin film (∏_1_ → 0), we get *q_LR_*=*q_Lc_*=*π γ ρ_L_ h_L_* /(*ρ_Q_ h_Q_*) and the formula for the frequency shift Δƒ*_0L_* as follows:

(20) $$\Delta f_{0L}=-\gamma f_{0}\frac{\rho_{L}h_{L}}{\rho_{Q}h_{Q}}$$

which corresponds to the Sauerbrey's formula [15] except for the parameter *γ*.

For the case of thick (∏_1_ → ∞) and elastic (∏_2_ → 0) solid, we have:

(21a) $$q_{LR}=M\tan N$$

(21b) $$q_{LI}=0$$

Since *q_LI_*=*0*, *G_max_* is the largest and independent of the material property. Further, Equation (21a) implies that there can be an infinite number of values of the parameter *μ_L_* that produces a given value of *q_LR_*, because at *N* = (2*n*-1) *π /2* (*n*=*1,2,3*…) *q_L_* becomes infinite. However, the case of thick elastic material may not be encountered so frequently.

For the case of thick layer (∏_1_ → ∞) of Newtonian liquid (∏_2_ → ∞), we have *μ* =*ω_0_ η_L_*. When the liquid has a low viscosity (*η_L_* → 0), then the frequency shift Δƒ*_0L_* can be given as:

(22) $$\Delta f_{0L}=-\gamma f_{0}^{3/2}\frac{\sqrt{\rho_{L}\eta_{L}}}{\sqrt{\pi \rho_{Q}{\bar{c}}_{66}}}$$

which is the same as the one derived by Kanazawa and Gordon [16] except for the parameter *γ*. In case of the resonator application to the measurement in water solution, the density and viscosity of the solution affect the frequency shift. Muramatsu *et al.* [17] showed that the experimental results of the shift linearly increase with the raise of square root of solution density and viscosity in water and ethanol-water solution.

**Figure 2.**
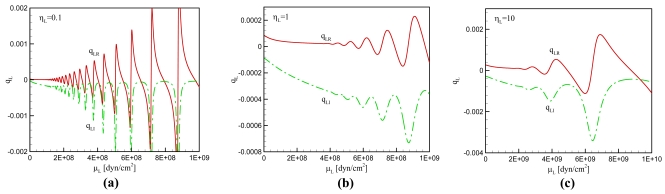
Sketch of a quartz crystal resonator with electrodes on both sides and a viscoelastic overlayer attached on the external surface of an electrode.

For the case of general viscoelastic overlayer, we can investigate the effect of *μ_L_* and *η_L_* on *q_L_* by plotting *q_L_* versus *μ_L_* at different values of *η_L_*. As shown in Figure 2, *q_L_* oscillates with *μ_L_* more pronouncedly at lower values of *η_L_*. A lower value of *μ_L_* produces the smaller amplitude of *q_L_* fluctuation. It again implies that we can expect multiple values of the parameter set (*μ_L_*, *η_L_*) which give rise to a given value of *q_L_*; selecting such parameter set is more highly possible at lower values of *η_L_* and *μ_L_*. From Equations (15) and (16) it is known that the frequency shift is determined from the real part of *q_L_* and the maximum conductance from the imaginary part of *q_L_*. When the experimental measurements of frequency shift and conductance are available, the estimation of *μ_L_* and *η_L_* is possible from the equations as demonstrated in Figure 2. In this case, we must pay attention to the possibility of multiple sets of the properties which has not been reported in the previous studies.

In the practical implementation of a resonator the viscoelastic overlayer is often loaded on the electrode surface. When a viscoelastic overlayer of polymer is applied to the resonator for the measurement of physical property variation along with a change of temperature or polymerization degree, the variation of viscosity and elastic shear modulus of the overlayer can be computed for the frequency shift and maximum conductance of the resonator. In other words, monitoring the frequency and the conductance can explain the polymerization and crystallization processes of a polymer sample. In this case multiplicity in the solutions of the equations may be resolved by a certain strategy. For instance, provided that the resonant property is known at a given parameter set, the viscoelastic property is readily obtained from the equations uniquely: When the experimental condition, such as temperature, varies continuously, the physical properties of overlayer also follow the condition and the determination of the properties from Figure 2 is a simple procedure.

## 3. Experimental

### 3.1. Materials

Polyethylene (Sigma-Aldrich Inc., St. Louis, MO, U.S.A.) having a number-average molecular weight of about 7,700 and a melting point of 90 °C was used as received. An AT-cut quartz crystal resonator having a base frequency of 8 MHz (Sunny Electronics Co., Korea) was utilized in this experiment. The electrodes of the resonator were silver finished.

### 3.2. Experimental Setup

**Figure 3.**
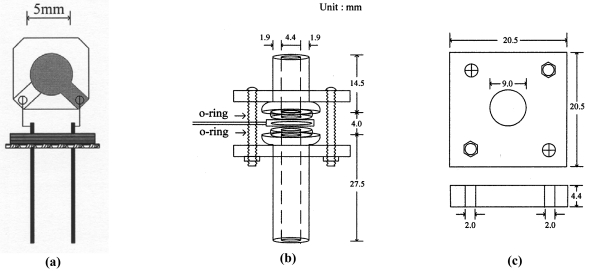
Schematic diagrams of quartz crystal resonator (a), resonator cell module (b) and holding bracket (c).

A schematic diagram of a quartz crystal resonator is demonstrated in Figure 3(a). The polyethylene overlayer was placed on the one of electrode surfaces of the resonator, and the resonator was heated and cooled in an oil bath. Because the resonator surfaces can not be in contact with oil for the accurate measurements of its resonant frequency, conductance and susceptance, the resonator was placed in a specially designed module. The cell module illustrated in Figure 3(b) was prepared with two glass holders, two O-rings, two bakelite brackets and four screws. The dimension of the brackets is shown in Figure 3(c). The resonator was placed between two glass holders, and two o-rings keep the oil from wetting the electrode surface. Two brackets tighten the glass holders with four screws.

**Figure 4.**
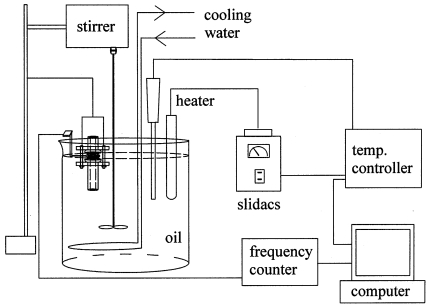
A schematic diagram of experimental setup.

The detailed dimension of the cell is given in the figure. After the module was assembled, fine particles of polyethylene were obtained by sieving the powder with a sieve of 250 μm. About one third of 0.1 mg of the polyethylene powder was placed on the top electrode of the resonator. The module was put in an oil bath demonstrated in Figure 4. For the better control of resonator temperature, the module was immersed to the level of the upper o-ring in the bath. The bath temperature was adjusted by electric heating, and water cooling was also provided for the cooling cycle in the experiment. The electric heater was controlled with a programmable temperature controller (Hanyoung Electronic Co., Korea, Model NP-200). The resonant frequency, resonant resistance and the temperature of oil bathwere measured using home-made devices, and an A/D converter was employed for signal processing. The digital signals of the resonant frequency, resonant resistance and temperature were provided to a PC for the data analysis.

### 3.3. Experimental Procedures

The experiment began at a temperature of about 25 °C. After the experimental setup was stabilized for an hour, the bath temperature was raised at a rate of 1 °C/min up to 100 °C, and was lowered at a rate of 1.5 °C/min. The first measurement was conducted at a temperature of 95 °C. At the temperature the setup was steadied for two minutes, and the measurement was conducted for 8 minutes using an impedance analyzer (Agilent Technologies, U.S.A., Model 4192A). The conuctance and susceptance were determined at a resonant frequency between 794 MHz and 804 MHz in a step of 50 Hz. The temperature was lowered by 4 °C down to 55 °C, and the measurement was done in the same manner

## 4. Application to Polyethylene Crystallization

The formula and analysis presented so far are applied to the experimental study on the measurement of material properties of polyethylene during its crystallization process. As the material and geometrical constant for the quartz, we consider the following parameters many of which are commonly used for an AT-cut crystal:

$$c_{66}=2.947\times 10^{11}\text{dyn}/cm^{2}$$

$${ɛ}_{22}=40\times 10^{-14}F/cm$$

$$\rho_{Q}=2.651g/cm^{3}$$

$$K^{2}=7.74\times 10^{-3}$$

$$h_{Q}=0.0205449\;cm$$

Here, the depth of quartz *h_Q_* was obtained in such a way that the resultant resonant frequency matches with the measured one *ƒ_0_* = 7,996 [*kHz*] without any overlayer. Further, we consider the following properties for silver electrodes:

$$h_{e}=30nm$$

$$\rho_{e}=10.5\times 3\times 10^{-5}g/cm^{2}$$

Then, we have *q_e_* = 0.00594. On the other hand, Equation (13) gives *ƒ_00_* = 8,114 *kHz* and we obtain the theoretical prediction for *ƒ_0_*, the resonant frequency without the overlayer, from Equation (12) as follows: *ƒ_0_* = 7,997.7 *kHz*. On the other hand, the computation with Equation (11) gives *ƒ_0_* = 7,996.0 *kHz*. We can see that the error of the theoretical prediction is only 0.02%. The electrode diameter *d_e_* takes different value for each experimental measurement to match the measured *G_max_*Δƒ_12_ using Equation (17).

**Figure 5.**
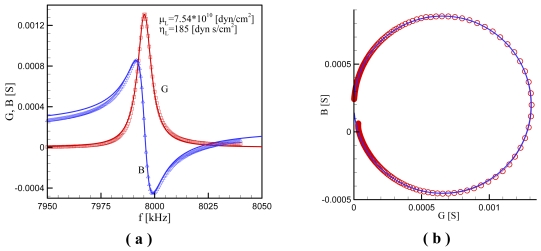
Comparison between calculated (lines) and measured (symbols) conductance (G) and susceptance (B) at *T* = 83 [°C] (a) in the ƒ – *G* and ƒ – *B* planes; (b) in the *G* – *B* plane (Nyquist plot).

**Figure 6.**
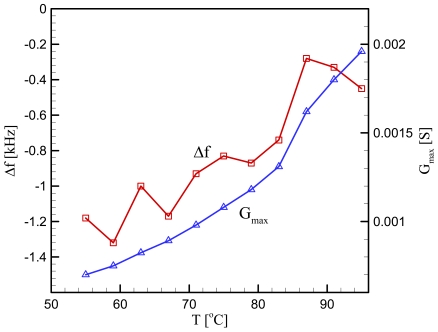
Experimental results of Δƒ and *G_max_* obtained at 11 different temperatures.

The experiment provided the conductance *G* and the susceptance *B* as a function of the frequency *ƒ* for the case without overlayer at the room temperature and 11 cases with melted polyethylene at different temperatures, and a typical set of data at a temperature of 83 °C is shown in Figure 5(a). First, for the unloaded case, we read *G_max_* from the *G* curve of Figure 6(a) or from the radius of a circle matching the Nyquist plot as shown in Figure 5(b), and also read Δƒ_12_ from the *B* curve in Figure 5(a). From Equation (17) we calculate the electrode diameter; *d_e_* =0.5101*cm*. Then we find ξ from matching *G_max_* obtained from the experiment with that given by calculating (16). Equation (17) can be used as a guide or initial value for ξ, where *R_m_* =1/*G_max_* is applied. Equation (17) yields ξ = 2.08 × 10^-4^, whereas the numerical calculation gives ξ = 2.12 × 10^-4^ being only 2% different from the asymptotic calculation.

**Figure 7.**
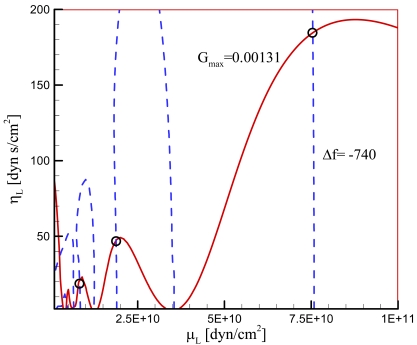
Typical map for determining *μ_L_* and *η_L_* of a viscoelastic overlayer. Solid lines are contour lines for Δƒ = −740[Hz] and dashed lines for *G_max_* = 0.0013[S] at 83 °C.

**Figure 8.**
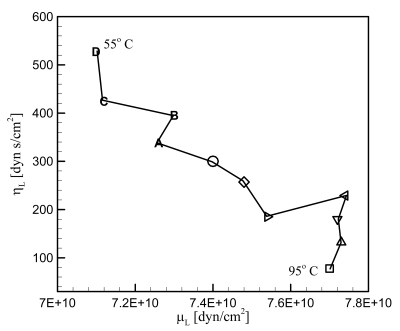
Estimated values of *μ_L_* and *η_L_* of a viscoelastic overlayer at each temperature with fundamental mode of oscillation. The temperature starts from 95 °C decreasing down to 55 °C with 4 °C increment.

We next obtain the parameters *μ_L_* and *η_L_* for loaded cases. The measurement was done with the PE particles melted on the electrode surface. The total mass of the particles is 0.033 mg. It is assumed that all the particles contribute to a single particle, its size being determined by summing up all the particles' size; *d_L_*=*0.0502 cm*. This in turn provides the thickness of the PE particle; *h_L_*=*0.0184 cm*. Since the melted particles do not change their shape with temperature, *d_L_* and *h_L_* are fixed in the subsequent calculations. It is true that a parameter set (*μ_L_*, *η_L_*) results in one set of parameters *q_LR_* and *q_LI_* and therefore one set of values *G_max_* and Δƒ. However as analyzed in the previous section, the inverse is not true. In fact, infinite number of parameter set (*μ_L_*, *η_L_*) can result in a given parameter set *G_max_* and Δƒ. Figure 6 presents *G_max_* and Δƒ measured from the experiment at 11 different temperatures. We see that *G_max_* decreases monotonously with the temperature decrease, but Δƒ shows fluctuation although in overall it also decreases with the temperature decrease. The sensitivity of the experimental setup is determined by the measurement resolution of the impedance analyzer. The resolution of frequency measurement is 1 Hz, and that of conductance is 1 nS. Therefore, the variation trends of the frequency difference and conductance in Figure 6 are real, while the fluctuation in the frequency is due to the instrumental sensitivity. To find the parameter set (*μ_L_*, *η_L_*) corresponding to each of the 11 data set (*G_max_*, Δƒ), we generate a map on the (*μ_L_*, *η_L_*) space as shown in Figure 7. Intersection of contour lines of *G_max_* and that of Δƒ then gives us the parameters *μ_L_* and *η_L_*. Figure 8 exhibits the parameters obtained in this way at the fundamental mode of the overlayer vibration. We see that as the temperature decreases from the melting point, the shear modulus decreases but the viscosity increases.

The measurements of elastic shear modulus and viscosity shown in Figure 5 are compared with those of bulk polyethylene in melt state [18], and the results are listed in Table 1. The top three rows are of the instrumental measurements in the low rate of shear, the estimated values of this study is followed, and the bottom row is the estimation for the high frequency viscoelasticity from the low frequency measurements. Because the melt properties are of a large difference of frequency, a direct comparison between the measurement and the estimation of this study is difficult. However, the two rows show comparable values of viscoelasticity. The tendencies of the increase of elastic shear modulus and the decrease of viscosity with the frequency elevation of sample movement show that the measurements of this study are same to the melt properties. In addition, the effects of temperature variation on the elastic shear modulus and viscosity are the same in the studies. While the instrumental measurement of rheological property is limited to polymer melt, the proposed measurement technique of this study can be implemented to the wide range of applications for various materials.

## 5. Conclusions

A generalized relationship between the viscoelastic property of a polymer overlayer placed on the electrode of a quartz crystal resonator and its resonant charateristic is developed from the mechanics of the quartz movement and applied to the determination of the viscoelastic properties during the polymer crystallization. The developed procedure is utilized in polyethylene crystallization, and the estimated viscosity and elastic shear modulus from the measurements of resonant frequency and conductance of the resonator are compared with those of a polyethylene melt determined instrumentally. The comparison indicates that the estimation is comparable to that of the melt, implying the possibility of wide application of the proposed technique in the field of polymer processing.

## Figures and Tables

**Table 1.** Experimental and estimated values of viscosity and elastic shear modulus.

**Frequency** (Hz)	**Viscosity (dyn s/cm^2^)**	**Shear modulus (dyn/cm^2^)**
Bulk [18]		
0.1	2 × 10^5^	1.5 × 10^4^
1	1.1 × 10^5^	1 × 10^5^
4	6 × 10^4^	1.5 × 10^5^
Overlayer		
8 × 10^6^	185	7.54 × 10^10^
From bulk data		
8 × 10^6^	346	6.47 × 10^9^
